# Reply to Tanaka and Kunieda: Control protein GFP also shows a mesh-like structure in desiccating tardigrade cells

**DOI:** 10.1073/pnas.2316451120

**Published:** 2023-11-20

**Authors:** Sae Tanaka, Kazuharu Arakawa

**Affiliations:** ^a^Institute for Advanced Biosciences, Keio University, Tsuruoka 997-0017, Japan; ^b^Exploratory Research Center on Life and Living Systems, National Institutes of Natural Sciences, Okazaki 444-8787, Japan; ^c^Faculty of Environment and Information Studies, Keio University, Fujisawa 252-0882, Japan; ^d^Graduate School of Media and Governance, Keio University, Fujisawa 252-0882, Japan

We recently reported an in vivo expression and live imaging technology for tardigrades and an unexpected tissue specificity of anhydrobiosis-related gene expression, with the CAHS3 gene, for example, being most intensively expressed in the epidermis of tardigrades (1). Using immunohistochemistry, Tanaka and Kunieda now provide interesting confirmation that CAHS3 is indeed localized in the epidermis but is also expressed in the storage cells (2). The authors then suggest careful consideration of the use of vector-induced expression based on this result. As we have shown in *SI Appendix*, Fig. S5, while SAHS1 is most intensively expressed in the storage cells, some expression was observed in the epidermis (1). This is consistent with the several-fold higher expression of SAHS1 and lower expression of CAHS3 in the storage cells as observed by our tissue-specific RNA-Seq (1), and this quantitative nature is another useful aspect of live imaging. Our previous work was based on observations at 48 h after vector injection with only a short cis-regulatory region, which should not be confused with the complex proteostasis of native proteins. On the other hand, we now have evidence that TardiVec is retained and functional for weeks, with low-contrast imaging showing weak expression of CAHS3 in storage cells, which would facilitate our understanding of protein turnover and maintenance in tardigrades. Nevertheless, we appreciate the confirmation of the tissue-specific expression pattern of CAHS3 by immunohistochemistry, with no observable signal in muscle or gastrointestinal cells.

One of the focuses of recent molecular studies of tardigrade anhydrobiosis has been the reversible filamentous gel formation of the CAHS protein, which we reported for CAHS1 (3), followed by several papers on different paralogs and orthologs (4–7) including that for CAHS3 by Tanaka et al. (8). In their commentary, Tanaka and Kunieda argue that filamentous structures are observable in desiccating tardigrade cells by immunohistochemistry (2), but we prefer to be cautious about such interpretations as we have previously reported, based on no observable difference from controls expressing mEGFP and mCherry (1). Our recent observations of anhydrobiotic tardigrades have provided a finer representation of tardigrade cells. In an epidermal cell from a control mEGFP-expressing tardigrade, we observed a mesh-like structure that appears to correspond to Tanaka and Kunieda’s (2, figure 2B) (Fig. 1). Based on our live imaging observations, the mesh-like structure can be attributed to the presence of multiple vesicles and cell compaction during desiccation. It would be interesting to see how other control proteins behave under immunohistochemistry, but the ease of using heterologous proteins for control is another advantage of vector-based live imaging.

**Fig. 1.**
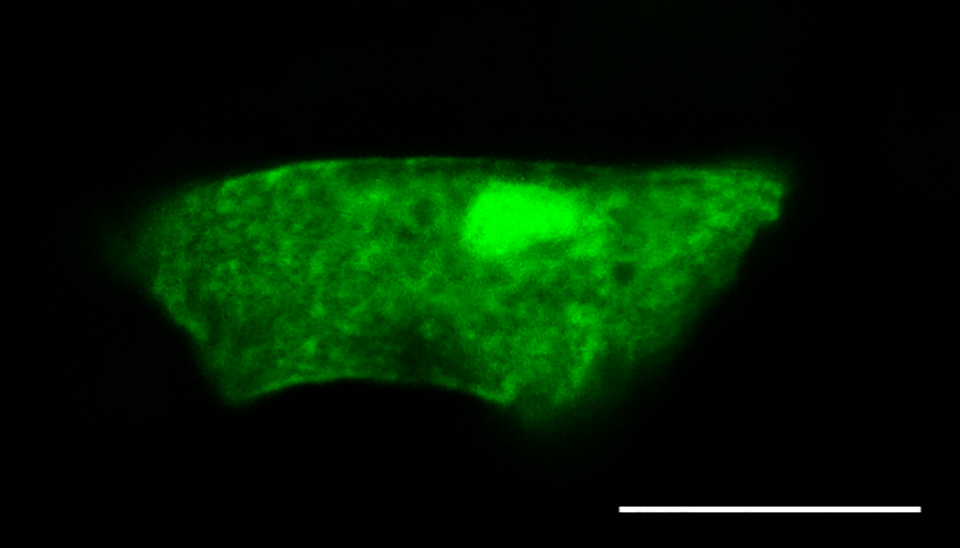
mEGFP showed a mesh-like structure in an epidermis cell of a tardigrade upon desiccation. A tardigrade introduced with pRvCAHS3-mEGFP vector was observed on a gelatin-coated glass for 30 min RT. (The scale bar is 10 µm.)

Direct observation in tardigrades is an important direction, with traditional approaches like immunohistochemistry facilitated by new techniques such as our vector system. In addition, newly developed methods such as expansion microscopy and spatial transcriptomics can provide a clearer picture of anhydrobiotic tardigrades.

## References

[1] S. Tanaka, K. Aoki, K. Arakawa, In vivo expression vector derived from anhydrobiotic tardigrade genome enables live imaging in Eutardigrada. Proc. Natl. Acad. Sci. U.S.A. **120**, e2216739120 (2023). 36693101 10.1073/pnas.2216739120 PMC9945988

[2] A. Tanaka, T. Kunieda, Considerations on the TardiVec-based analyses of tissue-specificity and desiccation-induced supramolecular structure of target proteins. Proc. Natl. Acad. Sci. U.S.A. **120**, e2312563120 (2023).3798350810.1073/pnas.2312563120

[3] M. Yagi-Utsumi , Desiccation-induced fibrous condensation of CAHS protein from an anhydrobiotic tardigrade. Sci. Rep. **11**, 21328 (2021). 34737320 10.1038/s41598-021-00724-6 PMC8569203

[4] A. Malki , Intrinsically disordered tardigrade proteins self-assemble into fibrous gels in response to environmental stress. Angew. Chem. Int. Ed. Engl. **61**, e202109961 (2022). 34750927 10.1002/anie.202109961 PMC9299615

[5] M. T. Veling , Natural and designed proteins inspired by extremotolerant organisms can form condensates and attenuate apoptosis in human cells. ACS Synth. Biol. **11**, 1292–1302 (2022). 35176859 10.1021/acssynbio.1c00572 PMC9651988

[6] J. E. Eicher , Secondary structure and stability of a gel-forming tardigrade desiccation-tolerance protein. Protein Sci. **31**, e4495 (2022). 36335581 10.1002/pro.4495 PMC9679978

[7] J. Eicher, B. O. Hutcheson, G. J. Pielak, Properties of a tardigrade desiccation-tolerance protein aerogel. Biophys. J. **122**, 2500–2505 (2023). 37149732 10.1016/j.bpj.2023.05.002 PMC10323019

[8] A. Tanaka , Stress-dependent cell stiffening by tardigrade tolerance proteins that reversibly form a filamentous network and gel. PLoS Biol. **20**, e3001780 (2022). 36067153 10.1371/journal.pbio.3001780 PMC9592077

